# Local ASF challenges: the Philippine perspective on ASF stamping-out policy

**DOI:** 10.3389/fvets.2025.1691490

**Published:** 2025-10-15

**Authors:** Harvie P. Portugaliza

**Affiliations:** Department of Veterinary Clinical Sciences, Faculty of Veterinary Medicine, Visayas State University, Baybay City, Philippines

**Keywords:** animal disease control, African swine fever (ASF), culling policy, depopulation policy, livestock policy, Philippines, rural livelihoods

## 1 Introduction

Since African swine fever (ASF) was introduced to the Philippines in 2019, the country's swine industry has incurred substantial economic losses and a nationwide deficit in pork and pork-related products (1). As a result, the prices of basic commodities have increased, pushing minimum wage earners and 89% of small family farmers beyond the poverty line (2, 3). While high mortality rates caused by ASF explained the decline in the pig population, the enforcement of the stamping-out policy to control its spread may have dramatically contributed to the overall population decline (4, 5).

In the context of this article, stamping out refers to the depopulation of pigs, both from ASF-confirmed and potentially infected herds, within a defined infected zone to prevent disease transmission. In theory, this approach can rapidly contain outbreaks when implemented effectively (6, 7). However, its success relies on preconditions, including early outbreak detection, rapid response capacity, effective compensation mechanisms, and strict biosecurity compliance, all of which are often deficient in low-resource settings (8, 9). While stamping out based on zoning is recommended by the World Organization for Animal Health (WOAH) and has been shown to be effective when combined with other disease control strategies in some high-income countries (10), its implementation in low- and middle-income countries (LMICs) has been fraught with challenges in terms of financial and human resources and farmers' acceptability (11, 12).

In the Philippines, outbreaks of ASF have highlighted the limitations of current policies for controlling transboundary animal diseases. This issue is particularly significant for smallholder pig production systems, which account for the majority of the country's pig sector (13, 14). Experiences in the Philippines suggest that implementing a blanket stamping-out policy may raise concerns among smallholder farmers, who are among the most vulnerable stakeholders during ASF epidemics. The primary objective of this article is to examine the challenges faced by the Philippines, particularly smallholder farmers, in implementing a stamping-out strategy, while also exploring potential approaches to address these challenges for comprehensive ASF control. Although the Philippines is used as a reference case, the broader issues of compliance, biosecurity, and stakeholder engagement may also be relevant to other LMICs with traditional pig production systems.

## 2 Key components of the stamping-out policy

The Terrestrial Animal Health Code of WOAH defines stamping out as a policy designed to eliminate animal disease outbreaks. This policy is implemented by an authorized veterinary agency, which refers to the governmental body of the member country holding the relevant mandate (15). In the Philippines, this authority is represented by the Department of Agriculture (DA). Stamping out has long been a fundamental component of a strategy for controlling transboundary animal diseases. When executed swiftly and comprehensively, it can effectively halt the spread of ASF and allow a country to regain its ASF-free status, provided that it is combined with early disease detection through active disease surveillance, outbreak investigation and tracing, quarantine and control of animal movement, and strict biosecurity measures (4, 16).

Stamping out can be classified into three primary components. First, it involves the humane culling of animals in a herd with confirmed disease, including those that have been exposed to infected animals within the infected zone, even in the absence of clinical signs. It also extends to other herds that have been exposed to infection, whether through direct or indirect contact, based on a defined measure of distance. In the Philippines, this policy has been outlined through a series of government issuances by the DA (Table 1) and is mainly governed by the DA-Regional Field Office and the Bureau of Animal Industry (BAI), in coordination with the Local Government Units (LGUs). Here, stamping-out policy is executed over a maximum period of 5 days within a defined radius from the infected farm, encompassing infected and exposed herds. The stamping-out radius is within a 500 m or 1 km, following a modified 1-7-10 zoning protocol. Second, the policy involves the proper disposal of carcasses, including animal products, through rendering, burning, or burial, as applicable. The preferred method in the Philippines is burial within the farm or premises. Lastly, the stamping-out policy requires a decontamination phase, which involves cleaning and disinfecting premises using methods tailored to the specific causative agent (15).

**Table 1:** Issuances from the Philippine Department of Agriculture (DA) relating to the ASF stamping-out policy.

**Issuance type**	**Date**	**Title/Purpose**	**Provision on stamping-out policy**
DA Memorandum Circular No. 10	September 10, 2019	Veterinary quarantine movement protocol and reiteration of food safety measures during animal disease outbreaks/emergencies	Implementing 1-7-10 protocol, with mandatory tests and destruction of animals within 1-km radius (quarantine area) of infected area.
DA Memorandum Circular No. 32	December 2, 2019	Enhancing animal disease reporting and control through provision of compensation for swine culled during government-organized depopulation	Incident command for ASF control is mandated to include culling and compensation operations.
DA Administrative Circular No. 12	December 10, 2019	National zoning and movement plan for the prevention and control of ASF	Establishing 1-7-10 zoning protocol in ASF infected provinces and corresponding movement protocols.
DA Administrative Order No. 22	May 29, 2020	Guidelines on swine depopulation after ASF confirmation	Providing options on selective and non-selective depopulation in the quarantine area, which is now within 500-m radius of infected area.
DA Administrative Order No. 7	February 8, 2021	Implementing guidelines for the “Bantay ASF sa Barangay” program.	Protocol within 500-m radius of infected area: ASF-positive animals will be stamped out (Test and Destroy), while ASF-negative animals will be slaughtered for consumption (Test and Slaughter).
DA Memorandum Order No. 28	March 8, 2021	Submission of laboratory and depopulation reports for ASF	Monitoring depopulation count data.
DA Administrative Order No. 10	August 15, 2024	Revised guidelines for granting cash assistance to hog raisers affected by the African swine fever (ASF)	Cash assistance for farmers affected by ASF and the stamping out policy.

## 3 Practical challenges of the stamping-out policy

In high-income countries with industrialized livestock systems, stamping out is supported by rapid diagnostics, a robust veterinary infrastructure, and reliable compensation schemes (17–19). Therefore, implementing the policy is resource-intensive and requires full governmental support, as the financial implications can be substantial. According to data from 2020 presented by Casal et al. (20), US$32 million of the economic impact of ASF in the Philippines is attributable to the stamping-out efforts, which accounted for 55% of the total US$58.7 million expenses or loss. On average, this translates to stamping-out costs of around US$1,772 per outbreak. Its financial implication is not limited to the Philippines, with Vietnam spending 89.1% of its total outbreak costs in a similar year (20).

The amount of funding needed to mobilize a stamping-out plan in a single outbreak raises questions about its practicality in a resource-constrained country. In LMICs, where veterinary services are underfunded (21), the logistics of depopulation can lead to delays, errors, and even incomplete implementation (12). In most provinces, ASF may have spread silently due to insufficient surveillance and non-reporting of cases (22, 23). By the time an outbreak is officially confirmed, it may have extended beyond the initial foci. Hence, it prompts a crucial question: is implementing stamping out within infected zones effective in controlling outbreaks, considering delays in case reporting and even non-reporting, confirmatory diagnoses, and the deployment of responsible personnel?

Underreporting, as well as delayed reporting, can be traced back to farmers' reluctance to report ASF cases due to financial losses and the inadequate implementation of government surveillance against ASF (22, 24, 25). Based on local observations, most official reports of outbreaks are likely to occur after ASF has spread uncontrollably, as evidenced by the massive deaths of pigs across several backyard farms that cross barangay borders before the authorities officially declare an outbreak. There is also a significant time delay from the moment clinical ASF appears in the field to when a government laboratory produces a confirmatory test result (26). In the Philippines, stamping out can only be executed based on confirmed laboratory results, either from the Regional Animal Disease Diagnostic Laboratory (RADDL), the national Animal Disease Diagnosis and Reference Laboratory (ADDRL), or BAI-accredited laboratories (27). On average, each RADDL is responsible for one Philippine administrative region, which typically includes approximately two cities, 19 municipalities, and 2,540 barangays. Some of these locations can be as far as 140 kilometers away from the laboratory (28). The number and distance could raise concerns regarding the timeliness and responsiveness of the laboratories during widespread outbreaks, with a reported waiting time to receive an ASF confirmatory result ranging from 5 to 7 days (26). Moreover, once stamping out begins, several issues can arise regarding the lack of adequate disposal sites, inconsistent enforcement of quarantine measures, and other logistical limitations that further impede the effectiveness and practicality of the policy. The shortage of veterinarians within LGUs, who play a crucial role in disease control efforts, only exacerbates these challenges (29).

Another concern of the stamping-out policy is the issue of compensation. The Philippine government offered indemnities for culled pigs, but payments have often been delayed and insufficient. The situation has likely created widespread disincentives among farmers to report suspected cases. This feedback has prompted the government to revise its guidelines for granting cash assistance to ASF-affected farmers (DA Administrative Order No. 10, series of 2024). While budgetary constraints hinder implementation of indemnification, the operation of livestock insurance through the Philippine Crop Insurance Corporation (PCIC) has proven helpful during ASF outbreaks. However, coverage among farmers is still below the total farmer population, with an estimated 40.7% coverage in 2023. The fear of uncompensated losses has led to the concealment of outbreaks or the clandestine sale of sick animals, in hopes of recouping lost capital, which directly contributes to the further spread of the disease (14, 24, 25).

## 4 Acceptability of the stamping-out policy in rural communities

**Figure 1:**
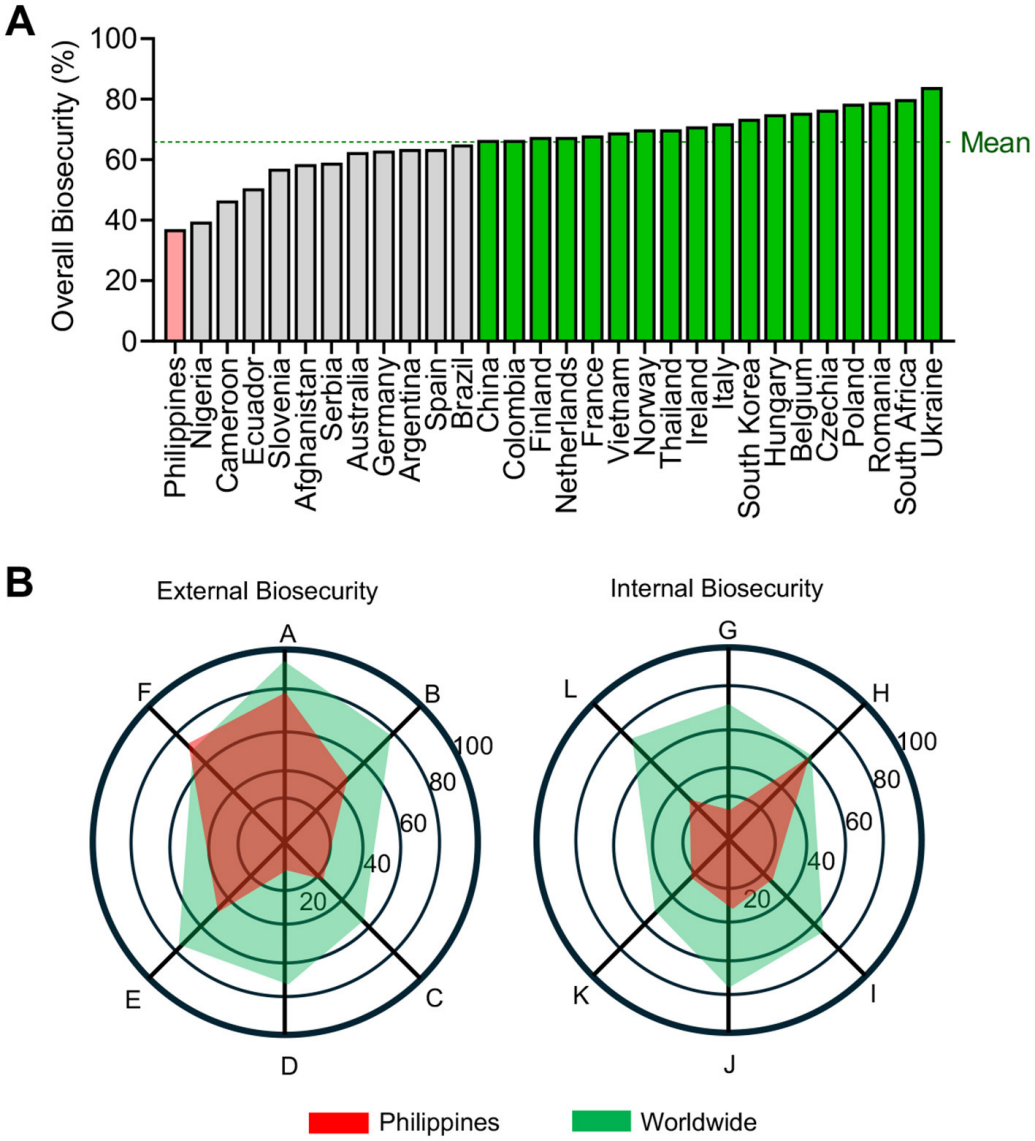
Biosecurity compliance of indoor pig farms in different countries, with real farm inputs recorded by Biocheck.Ugent accessed on August 8, 2025. **(A)** The country-level of overall biosecurity compliance, with a global mean value of 65.9%. **(B)** External and internal biosecurity subcategories showing the global and the Philippines' average data for each component. *Legend:* A - Purchase of breeding pigs, piglets, and semen; B - Transport of animals, removal of carcasses, and manure; C - Feed, water, and equipment supply; D - Visitors and farmworkers; E - Vermin and bird control; F - Location of the farm; G - Disease management; H - Farrowing and suckling period; I - Nursing unit; J - Finishing unit; K - Measures between compartments, working lines and use of equipment; L - Cleaning and disinfection. 29,688 total responses, with 2,634 responses from the Philippines.

In the Philippines, around 68% informal smallholder backyard farms operate in resource-limited environments (30). Backyard farms often lack biosecurity measures, which are crucial for controlling the spread of ASF (9, 31). The 2,634 responses from the Philippines on the Biocheck.Ugent indicates that indoor pig farms in the country have the lowest overall biosecurity compliance compared to 29 other countries (Figure 1), making farmers vulnerable to the continuous threat of ASF.

Raising backyard pigs holds cultural significance that extends beyond mere commodities. They are viewed as household assets and a form of savings that require minimal investment, often playing a role in community traditions (32). While backyard farmers assign value to their pigs based on subjective factors that exceed monetary measures (31), in most households, backyard pig rising is also a primary source of income (33). As a consequence of this perspective, farmers view the culling process negatively, particularly when animals are not exhibiting clinical signs (25), which can lead to potential resentment, mistrust, and trauma within the farmers' community (14).

When the stamping-out policy was implemented in several municipalities in the Visayas, anecdotal accounts indicated that farmers were quick to relocate their pigs to the mountains for concealment. In some cases, surviving pigs were sold to opportunistic buyers at a very low price to avoid total loss of capital. A few farmers were also adamant in rejecting the authority when told that their pigs would be included in the stamping out. These responses may reflect perceptions of inadequate financial assistance, unclear policies on live pig and pig product movements, a lack of defined recovery pathways, and limited support for transitioning to alternative livelihoods (34). Such perceptions are likely linked to insufficient engagement with farmers, where trust-building, clear communication, and the inclusion of stakeholder perspectives could have helped reduce resistance. The lengthy ASF bans on restocking, which can last over a year depending on compliance related to zoning status (Table 1), have also been affecting communities dependent on pig farming.

## 5 Beyond stamping-out policy in the Philippines

In the Philippines, many reported instances of ASF transmission have been associated with human behavior, including pig-raising practices, pig movement, and activities related to buying and selling live pigs, pork, and pork products (35, 36). The spread of ASF across administrative borders may reflect limited stakeholder awareness, or, at times, non-adherence to protocols, as suggested by reports of swill feeding, improper disposal of dead pigs, retention and sale of surviving pigs from affected herds, and animal movements despite zoning restrictions (22, 24, 25). These behaviors, however, are closely intertwined with poverty and marginalization, as many backyard farmers lack access to formal education and depend almost entirely on small-scale pig production, which may inadvertently sustain practices that increase disease risk (11, 37).

ASF transmission has therefore been facilitated, at least in part, by human actions, underscoring the expectation that community engagement and trust-building initiatives could play a crucial role in future control efforts (22). Local government units (LGUs), which are mandated to represent and serve their communities, may be well-positioned to lead such initiatives. When adequately supported, LGUs could help ensure that policies with substantial socio-economic consequences, such as stamping out, are communicated more effectively, while also providing space for dialogue on prevention and recovery strategies. The introduction of such policies is most likely to succeed when based on transparent communication and inclusive planning with local leaders and backyard farmers (38). Nonetheless, this approach has not yet been systematically evaluated in the context of ASF control, which represents a notable research gap in the Philippines. It is worth noting, however, that long-term strategies for other infectious diseases, such as foot and mouth disease (FMD), have included elements of community engagement in disease management. Such experiences suggest that community participation may have contributed to the eventual eradication of FMD in the Philippines, despite the application of stamping-out measures being limited to only a partial extent (39).

While stamping out remains an established tool for ASF control, its uniform application may present challenges in resource-limited settings. Some studies suggest that ASF control could benefit from context-sensitive strategies that include risk-based surveillance, selective culling, movement controls, and enhanced biosecurity practices (4, 12, 40). We expect that alternative approaches, if carefully designed, might also help safeguard indigenous farming systems that form part of the country's cultural identity. This could extend to efforts aimed at conserving local pig breeds and endemic wild pig populations, areas where the blanket application of the current stamping-out policy may face limitations in terms of feasibility and acceptability (40, 41).

The Philippine “Bantay ASF sa Barangay” program has outlined selective culling options across domestic pig production systems through “Test and Destroy” and “Test and Slaughter.” At this stage, these approaches appear more recommendatory than prescriptive, with limited operational detail available. Here, we discuss potential considerations that could inform their future implementation, while acknowledging the need for further policy refinement and field validation. The “Test and Destroy” policy is intended for ASF-positive pigs, allowing ASF-negative pigs to remain in the herd as assets and enter the food chain, potentially mitigating deficits in pork supply and reducing resource use compared to blanket stamping-out, as conducted in Vietnam (4).

The “Test and Slaughter” policy offers another alternative for ASF-negative pigs inside the infected zone, recommending that pigs undergo standard slaughtering procedures for human consumption in abattoirs with at least level “A” classification. Additional safeguards would likely be necessary to minimize transmission risks, such as processing pork (excluding offal), applying virus-deactivation methods (e.g., heating meat for ≥30 min at 70 °C) (15), and ensuring storage and distribution remain contained within the infected zone, or released only after testing processed pork for ASF. While a test-based culling system cannot fully overcome cultural and logistical challenges, it may help reduce resentment and resistance among farmers compared with blanket stamping-out, as previously reported in disagreements over the inclusion of apparently “healthy” pigs within affected zones (25, 42).

If supported by enhanced syndromic and risk-based surveillance with timely case reporting, one potential bottleneck may lie in the efficiency of confirmatory testing. A possible policy direction could be the decentralization of RADDL functions through the establishment of functional laboratories at the municipal and city levels. While this option may show potential, further evaluation is needed to assess its feasibility, sustainability, and effectiveness in strengthening ASF control in the Philippines. However, lessons from the COVID-19 pandemic highlight the critical importance of timely access to diagnostics and the value of expanding diagnostic capacity to reach even the most remote areas when developing preparedness and response plans (43).

Compartmentalization could also provide a viable option for sustaining international trade in subpopulations of pigs that adhere to strict biosecurity measures and are epidemiologically isolated from surrounding groups (44). While the stamping-out approach may restrict trade from affected regions, compartmentalization enables disease-free compartments to maintain exports of animals and products, as long as they comply with stringent surveillance, traceability, and biosecurity standards (18, 45). However, implementing and sustaining compartmentalization are primarily feasible for commercialized farms, notwithstanding that it can be challenging in resource-limited settings, where infrastructure and compliance capacity are often constrained (12). Thus, while it presents a viable opportunity to mitigate trade disruptions, its practical application for ASF control in the Philippines remains limited and requires substantial institutional and logistical support.

Lastly, improving biosecurity compliance remains a critical need in the Philippines for a holistic ASF control, particularly through innovations that adapt biosecurity protocols for backyard farming systems (35), or by gradually transitioning toward production systems where standard measures are more readily applied, such as medium- to large-scale farms (46). In small-hold settings, community-led biosecurity initiatives may be worth exploring as a means of encouraging shared responsibility among farmers, local leaders, and veterinary authorities (47). To support the harmonized application of biosecurity in scattered backyard farms, one possible strategy could be a cluster-based approach that organizes geographically close farms within a barangay. Such clusters might adopt cooperative systems to develop more standardized pig management practices and biosecurity measures. The government's role could focus on capacity development, farm registration, agricultural zoning, consistent outbreak surveillance, and engagement with farmers to lay down plans in case of an outbreak. Exploring alternative tools, such as zoning based on ASF risk levels and eventually ASF vaccination, could also yield more sustainable results.

## 6 Conclusion

The stamping-out policy has long been part of the Philippines' response to ASF. However, questions have been raised about its long-term practicality and social acceptability. In a country where pig farming is deeply ingrained in rural life and where veterinary systems continue to face challenges, stamping out the disease alone may not be sufficient; thus, the focus on disease control should be directed toward more comprehensive strategies. It may therefore be useful to consider complementary approaches that are pragmatic, community-oriented, and economically sensitive. Policymakers could aim to balance scientific rigor with socio-cultural realities, with the goal of ensuring that ASF control efforts do not inadvertently exacerbate rural poverty but instead support communities in moving toward more resilient and biosecure pig production systems.
